# Supplementary material for: Selective Loading and Variations in the miRNA Profile of Extracellular Vesicles from Endothelial-like Cells Cultivated under Normoxia and Hypoxia
Source: Int J Mol Sci. 2022 Sep 2;23(17):10066. doi: 10.3390/ijms231710066 (PMC9456085; doi:10.3390/ijms231710066)
Supplement: Supplementary file 1 [file ijms-23-10066-s001.zip › Sup_Fig_Manuscript_2022_IJMS_Reviewed_2.pdf]

Article

Selective Loading and Variations in the miRNA Profile of Extracellular Vesicles from Endothelial-like Cells Cultivated under Normoxia and Hypoxia

Anny Waloski Robert [1,†], Bruna Hilzendeger Marcon [1,†], Addeli Bez Batti Angulski [1], Sharon de Toledo Martins [2], Amanda Leitolis [1], Marco Augusto Stimamiglio [1], Alexandra Cristina Senegaglia [3,4], Alejandro Correa [1,4,*] and Lysangela Ronalte Alves [2,*]

Supplementary Figures

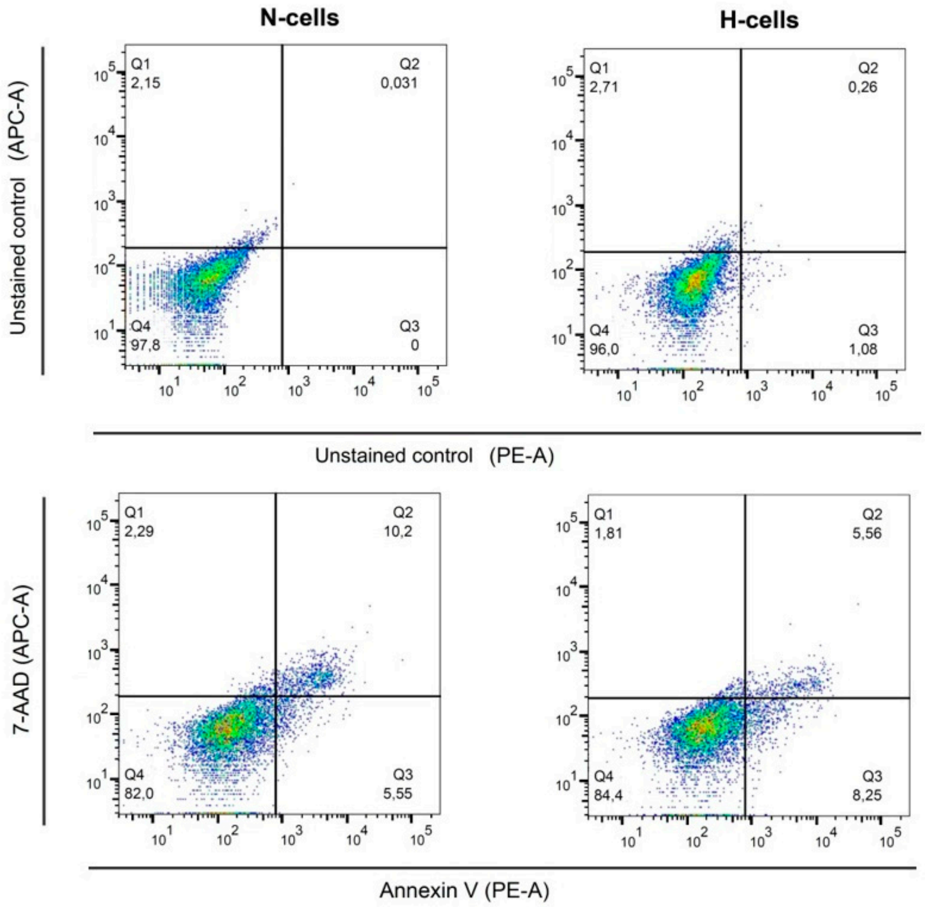

Figure S1. Apoptosis and necrosis assay of the E-CD133 1 cells cultivated for 72 hours under normoxic (N-cells) and hypoxic conditions (H-cells) for EVs isolation. Q1: Annexin V negative, 7-AAD positive cells; Q2: Annexin V/ 7-AAD positive cells; Q3: Annexin V positive, 7-AAD negative cells; Q4: Annexin V/ 7-AAD negative cell population. Dot plot quadrants were drawn considering stained cells in the permeabilized state (assay positive control; n= 1). Cells were incubated with PE Annexin V in a buffer containing 7-Amino-Actinomycin (7-AAD) and analyzed by flow cytometry. Unstained control were cells not incubated with Annexin V and 7-AAD. FACS analysis was performed on a FACSCanto II flow cytometer and data were analyzed using FlowJo software version 10.0.8r1.

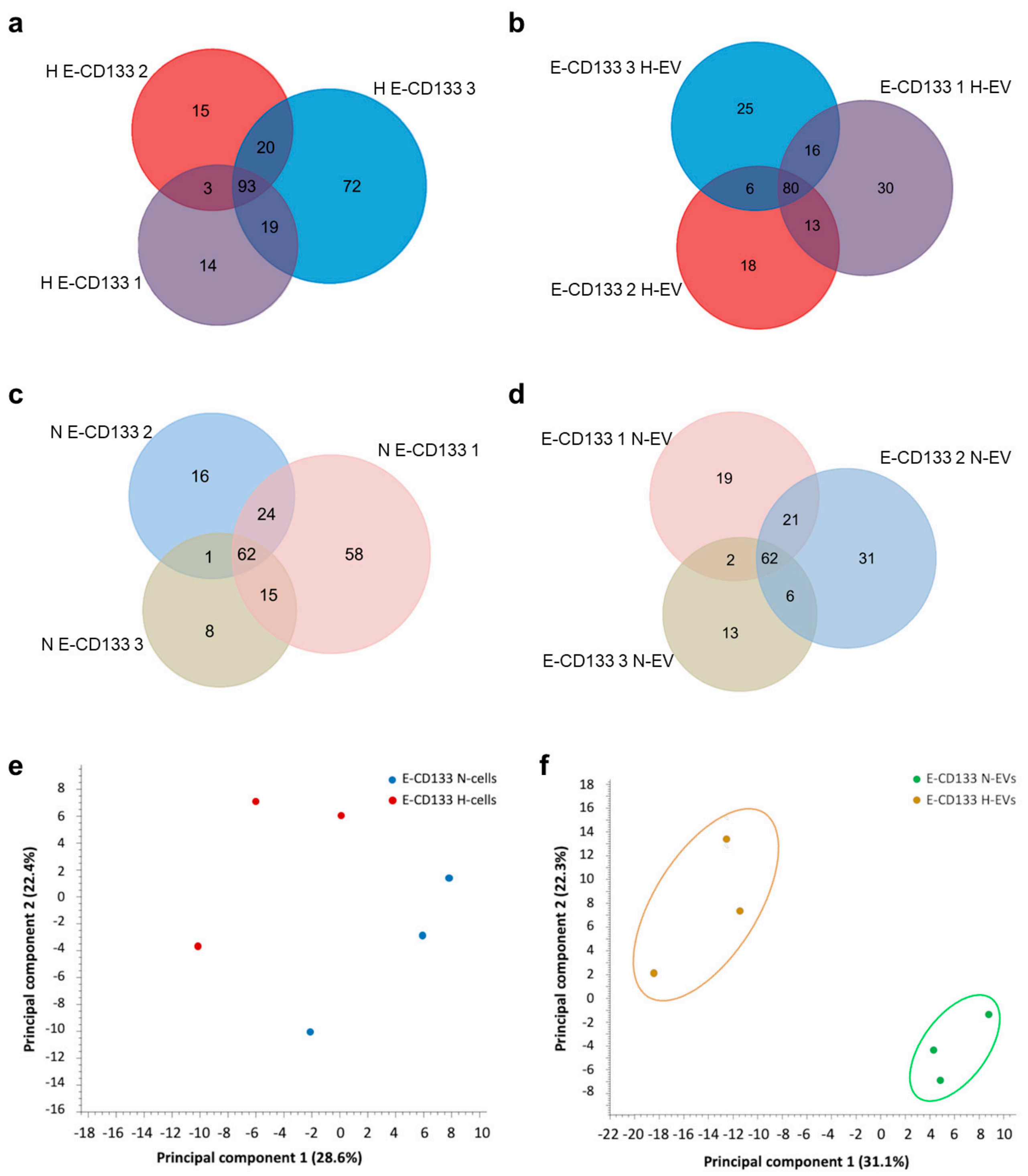

Figure S2. Comparison of miRNA identified in cells (E-CD133) and extracellular vesicles (E-CD133 EV) for each donor (1, 2, or 3). Venn diagram comparing miRNAs identified in cells (a) and EVs (b) derived from different donors, cultured in hypoxia. Venn diagram comparing miRNAs identified in cells (c) and EVs (d) derived from different donors, cultured in normoxia. (e) Principal component analysis of miRNAs identified in E-CD133 cells samples (normoxia and hypoxia). (f) Principal component analysis of miRNAs identified in E-CD133 EVs samples (normoxia and hypoxia).

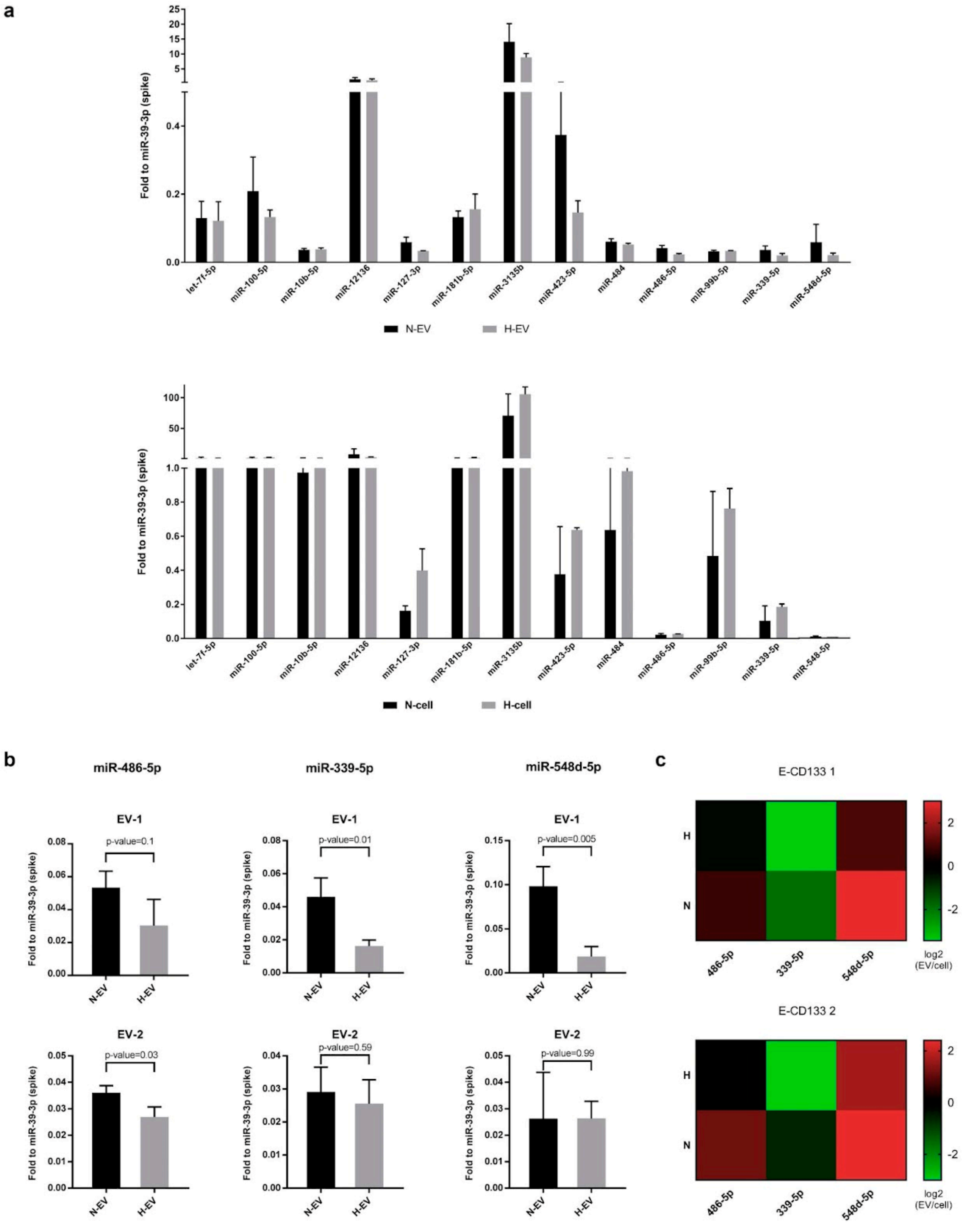

Figure S3: RT-qPCR analysis of miRNAs from E-CD133 EVs and cells during normoxia and hypoxia. (a) RT-qPCR of miRNAs identified in E-CD133 EVs (upper panel) and cells (bottom) (n=2 biological replicates, mean, standard deviation - SD). (b) The individual analysis of the RT-qPCR results obtained for each donor (E-CD133 EV-1 and E-CD133 EV-2) (n=3 technical replicates, mean, SD). Unpaired T-Test. (c) Analysis of the ratio of mir-486-5p, miR339-5p and miR-548d-5p expression between EVs vs cells. The heatmap shows the log2(EV/cell) for each donor (E-CD133 1 and E-CD133 2) in each condition (N and H).

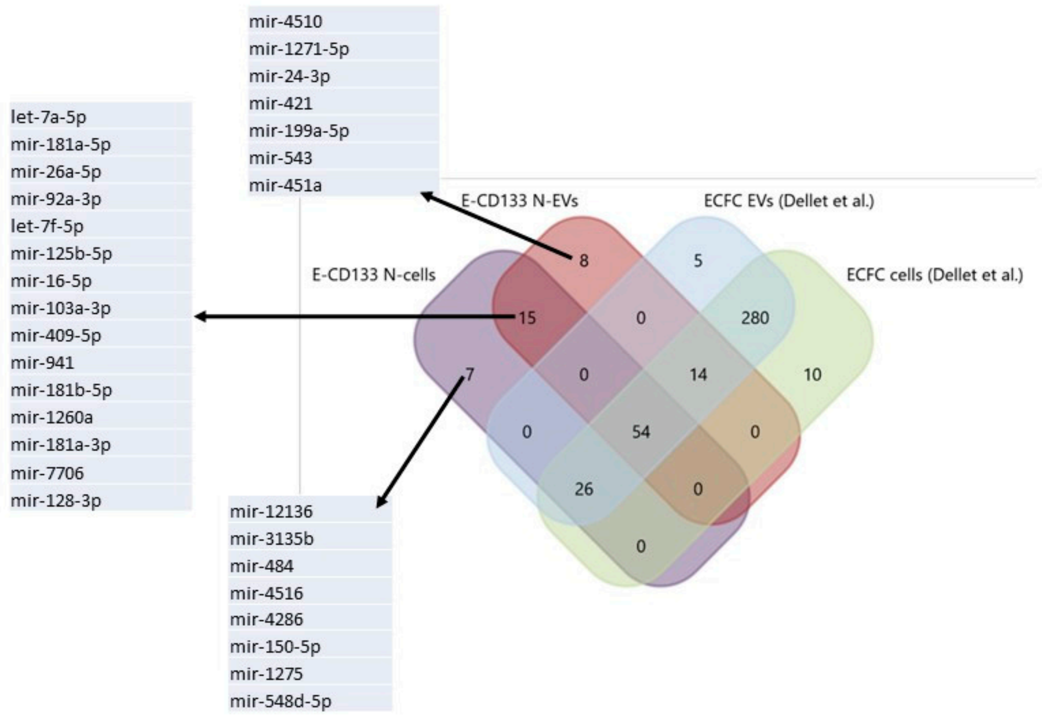

Figure S4: Venn diagram comparing the miRNAs identified by RNA-Seq in E-CD133 and ECFCs (Dellet et al., 2017) cells and EVs kept in normoxia.

a

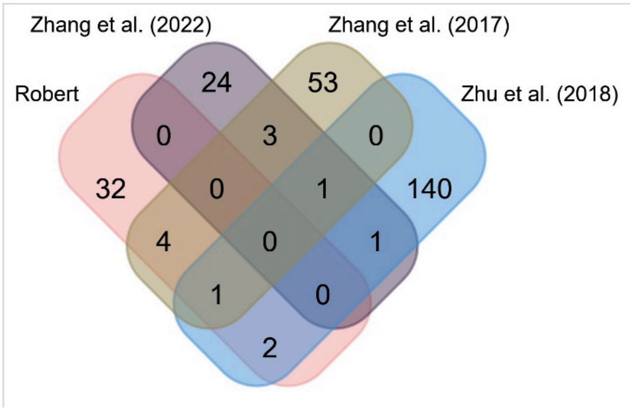

Zhang et al. (2022)
Zhang et al. (2017)
Robert
Zhu et al. (2018)
24
53
0
3
0
32
0
1
140
4
0
1
1
0
2

b

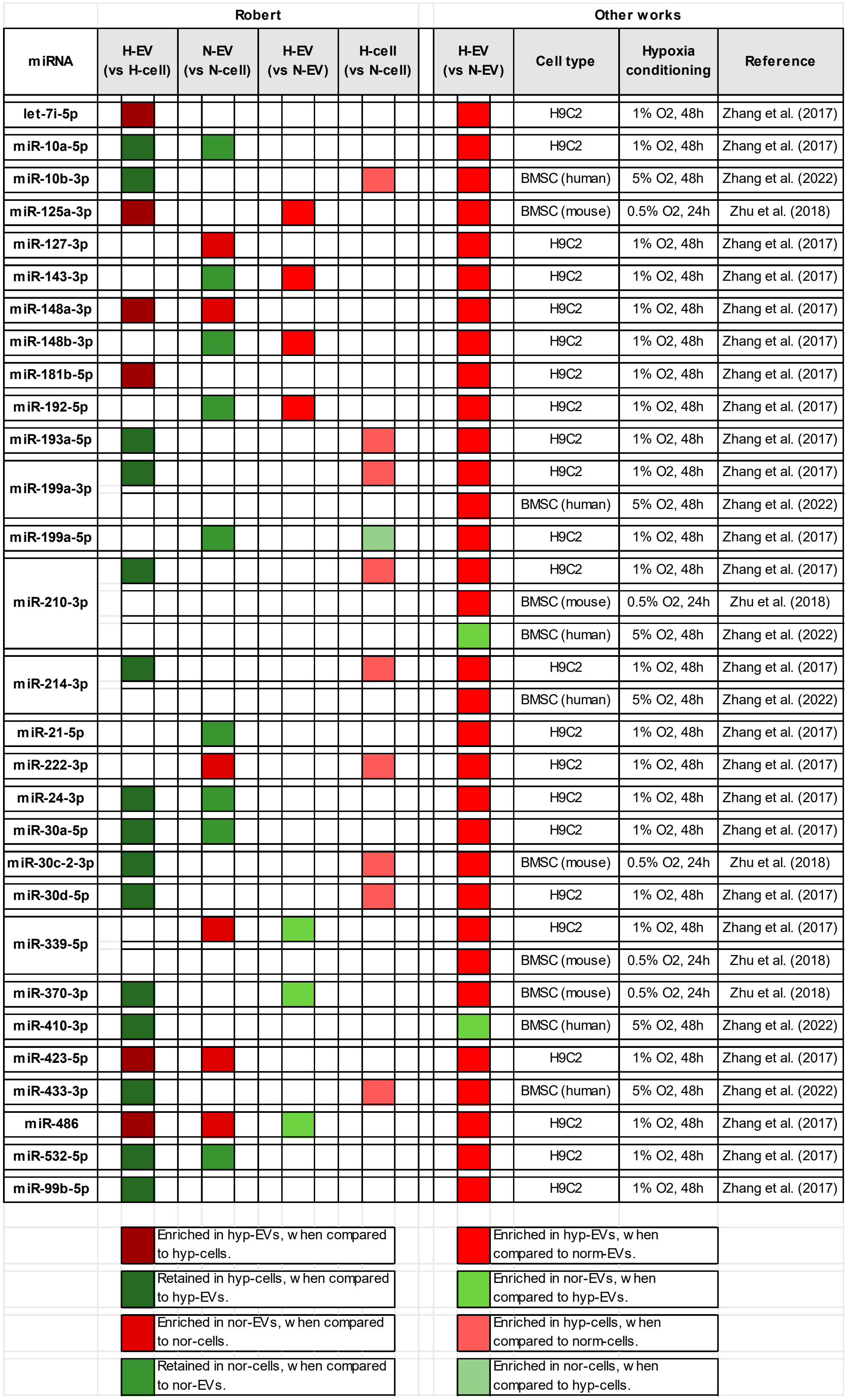

Figure S5. Differential enrichment of miRNAs in N-EVs and H-EVs. (a) Venn chart of the miRNA found as DE in N-EVs and H-EVs from E-CD133 cells, H9C2 (Zhang et al., 2017) and BMSCs from mouse (Zhu et al., 2018) and human (Zhang et al., 2022). (b) Analysis of the enrichment profile observed in our study of miRNAs found as DE in H-EVs vs N-EVs from E-CD133 cells, H9C2 (Zhang et al., 2017), and BMSCs from mouse (Zhu et al., 2018) and human (Zhang et al., 2022).
